# Exploring the unconventional: health professionals’ experiences into medication-free treatment for patients with severe mental illness

**DOI:** 10.1186/s12888-024-06251-8

**Published:** 2024-11-14

**Authors:** Elisabeth C. Klæbo Reitan, Henriette Riley, Tordis Sørensen Høifødt, Valentina C. Iversen, Anne Høye

**Affiliations:** 1 Division of Mental Health and Substance Abuse, University Hospital of North Norway, Tromsø, Norway; 2 Department of Clinical Medicine, UiT The Arctic University of Norway, Tromsø, Norway; 3 Department of Health and Care Sciences, UiT The Arctic University of Norway, Tromsø, Norway; 4 Department of Mental Health, Norwegian University of Science and Technology (NTNU), Trondheim, Norway; 5 Nidelv District Psychiatric Center (DPS), St Olav Hospital, Trondheim, Norway

**Keywords:** Mental illness, Medication free treatment, Psychosis, Employees experiences

## Abstract

**Background:**

In January 2017, the Norwegian government mandated the establishment of an inpatient unit for “medication-free treatment” for patients with severe mental illness at the University Hospital of North Norway in Tromsø. This study aims to explore the employees’ experiences with this unit.

**Method:**

Focus group interviews were conducted October 2021 – February 2022. For analysis, the participants were divided into three groups; S (staff working at the medication-free unit), M (people involved in management at the unit) and T (therapists working elsewhere in the hospital). The analysis followed the Systematic Text Condensation and interviews were recorded, transcribed and analysed using NVivo software.

**Results:**

Health professionals described their experiences with medication-free treatment through five main concepts: 1) Employees’ motivation; 2) Frames; 3) Network; 4) Relations; and 5) Patients’ motivation. Staff and management expressed strong motivation for an alternative to “treatment as usual,” focusing more on recovery and relationships than on the absence of medication. Therapists from other hospital areas highlighted resource allocation concerns and expressed a desire to learn from the unit. Challenges were acknowledged by all groups.

**Conclusion:**

The term “medication-free treatment’’ might be misleadning. While patiens at the unit can use medications, there is a strong emphasis on patient autonomy and the option to taper off medication and live a life without them. The study adds valuable knowledge about the the experiences of employees working at a medication-free unit, and provides insights into the complexity of treating severe mental illness, both with and without medication. It highlights the importance of sufficient time, stability and resources to focus on each patient’s strengths and challenges. All employees agree that tailored measures in long-term treatment and a clear focus on recovery should be integral, even without an emphasis on “medication-free treatment”.

**Supplementary Information:**

The online version contains supplementary material available at 10.1186/s12888-024-06251-8.

## Background

In 2015, the four regional health authorities in Norway were directed by governmental mandate to establish a medication-free treatment option for patients with psychoses [1]. Different models were thereafter applied in each of the four regions [2–5]. The Northern Norway Regional Health Authority, serving a health care region of 490 000 inhabitants [6], opted to establish a new six-bed unit from January 2017 situated at the University Hospital of North Norway in Tromsø [2].

National Norwegian guidelines support both medical and non-medical measures for treatment of psychoses [7]. Still, the governmental directive to all the health care regions sparked considerable discussion [8–13]. Justifiability of medication-free treatment was also addressed by The Norwegian Directorate of Health, in response to concerns about consent competence [14]. The joint association of user organizations (Fellesaksjonen) [15], which was the main advocate for this mandate to the health care regions, emphasized the freedom to choose what one believes in and wants (autonomy) [15].

However, Yeisen et al. describe the scepticism among several psychiatrists as to how the decision came forward, pointing at a lack of scientific evidence [11]. Many expressed their professional integrity as being independent of the instruction, which was experienced as unscientific and ideological. In the other Norwegian health care regions, the medication-free treatment was embedded in ordinary bed units, but also within these models there are conflicting aspects. Ødegaard et al. [8] describe the challenges the new policy posed for the therapists; the balance between what the patients wanted on one hand and treatment guidelines, resources and legal framework on the other. The therapeutic alliance is described as important and yet fragile. In Ødegaard et al.’s study on music therapy in particular [8], therapists’ reflections on medication-free treatment for patients with psychoses is also brought forward. Music therapy was often initiated when the patient did not want medication. The creative input by musical therapists is described as important to broaden the therapeutic “space”, leading to an increased focus on acceptance and possibilities.

Through interviews with milieu therapists, Beyene et al. [10] describe that to succeed with medication-free treatment, there is a need for a multidisciplinary, holistic approach with focus on each individual. They address that time and being a “professional companion” is important, and concludes that this requires what they call a humanistic rather than a medical paradigm.

The importance of professional integrity has hence been highlighted [11], and also the professionals’ role in ‘’helping patients to make changes in their life’’ [9]. Self empowerment and recovery are additional aspects, as illustrated through the flexible recovery model by Leamy et al. [16] in 2011 under the acronym CHIME (Connectedness, Hope and optimism, Identity, Meaning and Empowerment), the Recovery Colleges as described by Perkins [17], and Mueser et al.’s model of Illness Management and Recovery, which includes psychoeducation [18, 19].

What Yeisen et al. [11] put into words is the sceptisism among professionals, due to the common understanding that antipsychotic medication is a crucial part of the treatment of people with severe mental illness. This is in particular due to the risk of relapse linked to discontinuation [20, 21] but also to studies showing decreased all-cause mortality with use of antipsychotics versus no antiposychotic use in patients with schizophrenia [22, 23]. A study by Wunderink [24] concludes with better rates for recovery for those having had dose reduction/discontinuation of medication during early stages of remission after first episode psychosis, whereas most studies state the importance of medication continuity [21–23, 25, 26].

Following the Norwegian government’s directive, the Norwegian Institute of Public Health conducted a systematic review of the effectiveness of psychosocial interventions with and without medication, with the conclusion that no studies were found on psychosocial treatment without medication [27]. Another review investigated medication treatment per se [28]; two interesting studies from Finland were recognized but were not conclusive [29, 30]. Bola et al. suggested that the ability to respond to medication-free treatment without becoming psychotic, might indicate its potential effectiveness [30–32]. In a later study, Wunderink et al. addresses the need for individual adjustments to the the treatment content [33].

Studies on how shared decision-making works in practice for people with psychoses in Norway has been conducted by e.g. Haugom et al. [34, 35], emphasizing the need to focus not only on medication but also on other treatments, which are seen as supplementary.

We have earlier described patients’ motivation for applying for medication-free treatment [36]. In the present study, the overall objective was to explore health professionals' experiences with implementation of the medication-free treatment in a clinical hospital unit.

## Methods

### Setting

The medication-free treatment unit is physically and organizationally located within the Department of Mental Health and Substance Abuse at the University Hospital of North Norway in Tromsø, which is the largest city in the region [37].

The unit is aimed at adult persons with psychoses or bipolar disorder. The uptake area is the three northernmost Norwegian counties Nordland, Troms and Finnmark, which together covers 35% of the country’s area but populated with only 9% of the population [6]. Approval of admission is decided on the basis of the patients’ own application with a description of motivation, in addition to a referral from the general practitioner (GP). All admissions are voluntary, and network collaboration is emphasized. Despite the name, the unit provides medication when needed, but the intention is to offer an alternative to medication for patients with severe mental illness. The unit had been operational since 2017, providing study participants with real experiences to reflect upon in the interviews.

### Participants and recruitment

Employees from the medication-free unit and employees from other units at the hospital who did not work there, but had experience with patients receiving treatment there, were invited to participate during autumn 2021/ winter 2022 (additional file 1, 2).

All employees with at least one year’s work experience at the unit were invited, only a few that did not have the opportunity declined. The invitation was distributed to the staff by the unit manager. Professionals who did not work at the unit were personally contacted by the first author. These were invited to participate based on their relevant experience from having or having had patients being in treatment at the medication free unit. No one declined the invitation. The participants were grouped according to whether they were staff at the unit (S, *n* = 9), persons with management positions at the unit (M, *n* = 5) or therapists working elsewehere in the hospital (T, *n* = 6).

### Choice of method

A main focus in the unit is the interaction between patients and employees, but also interaction between employees. Furthermore, the professional/ideological discourse in the group of employees comes forward as a crucial basis for the unit’s practice. Focus group interviews were therefore chosen as an efficient method to collect data while at the same time observing and stimulating interaction between the participants. Group interviews also facilitate the researcher’s opportunity to increase the amount of data through creating an arena for discussion [38, 39].

The interview guide for the focus groups was developed for the present study (Additional file 3). In order to identify relevant elements, we deconstructed the interview guide for patients, presented in an earlier paper [36]. We chose these overarching topics: ‘’about the unit’’, ‘’ethics and possible dilemmas’’, ‘’working with the theme of medication-free-treatment’’, and open questions ‘’about themselves’’. The researchers’ evaluation after the first interview was that these topics inspired the participants in a satisfactory way.

Interviews were conducted in six groups (S = 3, M = 1 and T = 2) led by first and third author; psychologist and psychiatrist, respectively. Every interview lasted 1,5 h and there was one interview per group. In one of the interviews first author participated digitally, all other interviews were conducted physically. One interview was performed individually due to miscommunication about timing. Two participants withdrew (from the T group) due to lack of time, resulting in a total of 20 participants.

Informed consent was obtained from all participants, clarifying that data will be used in research articles (open access). The consent-form also state that data will be de-identified as far as possible (Additional file 2).

### Procedures for data analysis

The interviews were recorded, transcribed and transferred to the computer-assisted qualitative data analysis software NVivo 1.6.1 after de-identification. For analyses Malterud Systematic Text Condensation (STC) [40, 41] was used. Malterud’s method is particularly useful in health research, social sciences, and any field where understanding complex human experiences is essential. Malterud’s data analysis approach for qualitative methods, often associated with systematic text condensation, emphasizes a structured yet flexible way to analyze qualitative data. It involves several key steps: 1)Transcription and Familiarization: Gathering and transcribing focus group discussions to become familiar with the content, 2) Identifying Meaning Units: Breaking down the text into smaller units that convey significant meanings or themes, 3) Condensation: Summarizing these meaning units while preserving their core essence, leading to more manageable data, 4) Structuring and Interpretation: Organizing the condensed data into themes or categories, which allows for deeper interpretation and insights [40]. Malterud’s systematic text condensation method provides a comprehensive framework for qualitative data analysis. By blending rigorous analysis with a focus on participants’ experiences, it enables researchers to uncover meaningful insights that contribute to understanding complex social and health-related phenomena. Its structured yet flexible approach makes it a valuable tool for qualitative researchers [40].

## Results

The characteristics and work experience of the participants are displayed in Table 1.

**Table 1:** Description of participants (*N* = 20)

Characteristics	
Gender	14 women, 6 men
Age^a^	31–68 years (median = 48.5)
Occupation	*n*
Mental health nurse, occupational therapist, physical therapist, art therapist, clinical social worker or social educator	10
Specialist in psychiatry or clinical psychology	8
Peer support competence^a^	2
Other education	1
Work experience from mental health services^b^	*n*
< 10 years	4
> 10 years	16
Psychoses, emergency psychiatric care	15
Work support or peer support	3

^a^One also had another education

^b^14 at the medication-free unit, 6 from other units

**Fig. 1:**
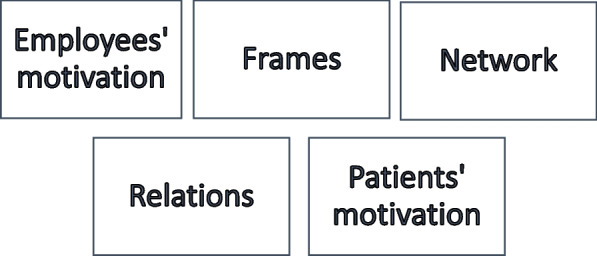
Main concepts for describing employees’ experiences with medication-free treatment

As shown in Fig. 1, exploring health professionals’ experiences with medication-free treatment reveals five main concepts which will be elaborated successively.

### Employees’ motivation

Motivation for working with medication-free treatment is by some linked to an overarching perspective on alternative ways to help people with severe mental illness. The importance of being able to help without “taking over”control and responsibility, and to reduce or remove shame, loneliness, isolation and stigma. One person explains motivation like this:

How to help without taking over … It’s in line with values that at least I am concerned about, being a human being, being professional … I feel I’m part of something important … The worst thing is not hearing voices or having delusions or anxiety. It’s outsiderness, stigma, shame, loneliness. These are things that happens in society and that concern people who have been severe ill. We often manage to create a community, lessening shame, stigma, loneliness, isolation. Because when we go together in network … it’s somehow antidotes to all these things (M)

Others express that the medication free unit represents an alternative understanding of illness and treatment, and words like ‘’paradigm’’ and ‘‘humanistic’’ are frequently used when describing the unit. Many of the participants express a need to distance themselves from a “biomedical model”, which might equal a biological model, and underline that the approach at the unit is something quite different, with a much stronger emphasis on individual, psychosocial elements than within “ordinary” psychiatry.

To a very small extent our understanding is in line with the medical model … the content is recovery-based in line with protocol (document on what the unit is supposed to do) and it’s relational and network-based. That is, it’s not about reducing or avoiding or even removing, but it’s about dealing with things in a way that is not an obstacle to life … It’s about us not understanding the symptoms exclusively as being inside the persons themselves but as something that happens in the context of relationships (M)

We’re concerned with how you understand this in relation to your life … being reactions to lives lived … a wide range of experiences of being human … As opposed to a chemical imbalance (M)

To give people the choice not to use medication is brought forward as essential, but it is also underlined that “medication free” has several nuances.

For many, the concept of “medication-free” is confusing, because we don’t say you have to taper down. They may use time tapering down. But, one of their main goals is to manage without or with as little medication as possible. The voluntariness is there; it’s me who chooses whether and at what pace I’ll go, but I will get help for it (S)

My impression is that they use time in advance talking about this and that they use very long time to taper down. Much longer time than patients’ want. And they do motivate them to keep some medication, too. So, the name is misleading (T)

When it comes to tapering down and to taking a break, it’s something we try to plan together with the network, using individual plans and (in dialogue) with the individual patient … We talk about that sometimes tapering off happens too fast, and one has to increase again. Sometimes one has to take a break. And that these are things we will talk about and find out along the way (clears throat) … If someone increases (medication dosage) again, it’s more often the patients themselves who recommend it rather than anyone else … If we can get a trusting relationship around this, we can talk about it in many ways (M)

People and relationships are described as the most important tools, also when discussions about medication is brought forward and the foundation for a positive relationship.

It feels good working with people who have applied for being here. Wanting this… (as opposed to earlier experiences) I’ve been much in contact with people who have not really wanted contact … (however now) they are at their most healthy … no emergency admissions… They are motivated for being here (S)

Several participants talk about focusing on ‘’life itself’’ rather than illness and diagnoses, and that tapering off medication demands a “holistic” approach.

We do not focus much on diagnoses (affirmative sounds by others). It’s not something lifted in meeting with patients. You meet them where they are and with whatever they bring of luggage and struggles. (Another adds:) They say that they appreciate this compared to other units. (S)

Before, I managed the other stuff. The less important, you know, helping to find a place to stay … (I’m) now working on life itself… We do talk about it (The other stuff like housing, money for food, occupation and medication compliance). It’s part of the whole package. And we do work on relations and network (S)

Medication is the lid that holds feelings and thoughts down, the reasons to take medication. When the lid is lifted, feelings and thoughts emerge and you become worse. Then you need a good framework around you, stability in treatment (and) with a therapist at home … That is why we use time, working towards the network … being stable and ready. Because when starting to remove medications, things start to move and it affects family, work, friends, children … Getting up in the morning, being able to participate in group meetings … It’s so important to work on what is happening on the inside, the feelings and thoughts … It’s very valuable to hear each other’s stories, to find support in each other (S)

### Frames

The concept encompasses several aspects such as agreed treatment context, legal framework, physical conditions and schedules, but also available resources and clinical dilemmas within the existing frames.

#### Context

Many express that it is hard work to be in treatment at the unit, with a fixed schedule from 8 am till dinner is done by 4 pm.

Being admitted to our unit is full time work. There is something going on all the time. They (the patients) often compare it to when they have been admitted to other units for mental health care, and have experienced that nothing happens apart from waiting for the next medication or a conversation (M)

Elsewhere there are no structures, nothing happens. (However) at our place there is (structure and things happening)! … We expect that they’ll get themselves up in the morning … and that they participate in what’s happening or according to their treatment plan … focusing on their own resources (S)

Being able to carry out the schedule they have made, is stressed as being important for how the medication free treatment works. This differs from what they experience elsewhere.

It sounds like it’s rocket science. It really isn’t. We do have structure, and then people wonder how we manage to get people out of bed in the morning. Well, perhaps we have a content that makes sense and that they want to participate in … nothing new, really (S)

This is also the impression as seen from some of the participants from other units.

It’s obviously a difference from ordinary bed units that the the treatment offered is more structured, and that they seem to manage to implement these treatment groups. We do try, too, but there is always something emergent that disturbs us (elaborates:) We want to do this in every unit, create structure (Another adds:) and we never manage to do that (T)

Structure is understood as transferable knowledge. That is; what works at the unit could also work elsewhere.

Many things they do could have been done more in other units – more structure and more activity (T)

I do think people get better if they have some content on a daily basis and learn to get up in the morning and to be part of what we plan to do … I believe this is transferable to other bed units. (T)

A clear structure is considered to make individual adjustments possible.

Patient care pathways … from the outside they look similar. But some are special and completely different from others ... What has fascinated me regarding structure is how we have been able to keep it … (talk about how implementation can fall apart if enthusiasts quit or are not present). At the medication free unit, it has always been … that we almost always manage to keep things (the schedule) going (M)

The unit is small and some receive extensive and long-lasting treatment. Having enough time is by many emphasised to be an important part of the therapeutic framework.

We allow people to take their time to find out if they are ready to taper down or reduce medication. Nothing is carved in stone. Some might need a few admissions to feel safe. (Another adds:) And some need long breaks from tapering down to stay stable. There has been people who were certain that they did not want to taper off, and then they are sluiced elsewhere (S)

Perhaps it’s like that elsewhere. However, my impression is that our relationship with time and clinical pathways, which we allow ourselves, not only allows us, but we do not know how long it will take (affirmative sounds from others). We have people who have been there (in a clinical pathway) for four years, and we still can’t say how long it will take. I think this is unique for a bed unit or when you work with the same patient for a long time (S)

Several of the participants underline that frames and content walk hand in hand, supporting each individual patient’s process.

The program delivers not only something to do, it can be useful if you taper down. Doing physical activites, getting customized with training programs from the physiotherapist … For the recovery workshop, perhaps you are not there to listen but to practice speaking with others, perhaps to set up some boundaries … to practice being together with people on a social arena. (Another adds:) An arena to learn to know oneself … Many do not know what they should work on when they arrive … (Another adds:) My experience is that many do not know themselves and have to learn to know themselves and who they are without medication. If perhaps years has gone bye and you have been sedated on medicines, you have forgotten who you were before and have to find out who you are (S)

Acknowledging that unit size might matter, they emphasize that it is the attitude towards structure itself that is important.

It might matter that we are few, of course, it’s easy to get an overview, it makes it safe to talk. But there are also the regular things we, the staff, do. Like having meals together with the patients … our structure is very flat. It has an impact on the feeling of power and powerlessness that at least patients feel in the building, it’s very low at our unit. They are for instance allowed to use the kitchen … There’s less the feeling of us/you than I think a larger unit can have problems with. (Another adds:) I think it’s about the attitudes we have … we’ve been talking about it, how we want things to be, and we still talk about it on our seminars … all the time … Off course, to have few patients gives us an oportunity to have this kind of structure … size matters but I really think it’s more about the attitudes we have (S)

Framework includes contexual factors related to organization of health services.

We cannot … (have) 6 people staying for long time if we are to be a realistic offer for the 480 000 people in the area. We have to be part of network having many patients at the same time. Throughout it’s a little above 30 active cases at the same time. Six admitted to the unit, the number varies, but about 30 in medication free treatment pathways (M)

The physical location of the medication-free unit, in the middle of a hospital mostly representing treatment as usual, can bring some expectations. Some of the employees from other units express that they had wished for larger synergy effects from having the unit inside the hospital, such as more systematic sharing of experiences and more active cooperation, both in relation to indivual patients and to treatment strategies in general. Nevertheless, they express having learned things that might have been less accesible if they had to learn them by just reading guidelines.

It’s been interesting getting to know how slowly tapering off is recommended, by now, at least, when the recommendations are so small dosages that we almost do not have so small dosages available … It’s possible that I would not have known this if the medication free unit was not close to us. Like taking a break for half a year when you are half way through if you have been using … relatively high dosage of antipsychotic medication. We could have been reading it from guidelines (T)

For some the location inside the hospital has also promoted the notion of not being too different from “treatment as usual”.

It’s been important how the unit has related to the rest of the hospital. It’s been important, and we’ve had an explicit goal of being part of the overall business. Not being too different and yet at the same time, not being so similar that the difference is not visible. It’s an idea from the beginning that the medication-free treatment offers throughout the country, here too, should influence the treatment in general. And I do think we have much left to do. And that’s partly how we think about it, being part of something more than our own unit (M)

#### Resources and justifiability

Framework includes ethical dilemmas concerning resources and justifiability. The rates of employees per patient are for instance much higher than elsewhere, and the patients must be quite well functioning to be admitted, even if they have a severe illness. They must also be able to consent.

There are few patients so the employees are capable of doing things (activities) … And it requires some shape, patients’ shape, to be able to cooperate (T)

I do think that if we are to work on the problem, neuroleptics do not solve it, they only alleviate the symptoms… you’ve got to staff up with enough resources … working on what lies behind. … handle the acting-out situations that will come. I don’t know if it’s the right thing for those who suffer the most. I do think you must be quite stable to manage it. I think it’s a balance between medication and being able to reach those patients who are receptive and want an alternative (T)

Perhaps it’s the way it should be, only 6-8- patients in a bed unit … I hear myself not dare to say (only) six … I try to tell myself that this is something we can learn from And what we have to challenge the authorities on. If you are acutely psychotic perhaps you shouldn’t be with more than 5-6 others (affirmative humming) to recover (T)

Many patients admitted to the unit function much better than any other patients in the hospital, but are admitted because the unit exists.

Somehow, I’ve been thinking, these are patients who do not function so badly that they would have been admitted if it hadn’t been for the medication-free unit. They would have been at home. Now they are in more active treatment and are admitted because of this (T)

Several participants, both staff working at the unit and outside, express a sense of exclusivity and uniqueness, and the increased level of resources is acknowledged as both present and, to some extent, problematic. Many are gratefulfor the possibility to work in a different way; something unique, within a large organization. Some express the higher level of resources spent as justifiable because of the content the unit delivers.

I find that they give more justifiable treatment than many other … other places there are too few resources and too much focus on medication (T)

I think they (the patients) are will be able to be there longer than I’m able to keep them … Regarding justifiability, it is ok because they can follow up, even offer readmissions. It would have been different if they didn’t do that … didn’t manage admissions and things like that, just disappearing into the emergency situations (at ordinary bed units) (T)

Some teams manage to “hold back”, whereas others are more impatient. This difference influences whether a patient will get admitted or not.

Someone thinks that everyone should be allowed trying, others don’t and find it’s useless to think like that. We do not discuss it together in a larger forum, just suddenly we are told that a new person will arrive and we hardly know what it’s about … The application goes to the manager group, they pass it on after an evaluation of whether it’s in our target group. It is to be further explored and you are given a team … Then it’s coincidental because some teams are clever at holding back whereas other are more impatient … We work on this (calibrating) (S)

Some express the skewed distribution of resources as unethical and frustrating.

At one meeting we were five doctors, it was absurd. Four psychiatrists and one doctor in specialization (laughs). It has made me think about the resources used … I do have other patients too that I wish could take part of these resources … I’ve felt a discomfort by someone getting so much … and others have got to settle with (little) … It’s painful on behalf of the patients. It’s the moral dilemma in mental health services. If you are exposed to a car accident, you’ll get five- six intensive nurses no matter what. Here it’s … random ... It’s unjust to the one not getting it. Ethically it’s ugly, I think (T)

General comments from staff outside the unit underline this sense of different resources distribution in hospital.

I think about it like a luxury unit, with money, it’s my fantasy, few patients ... not always filled up, many employees, hand picked. Newly established and everyone wants to work there. Calm, structured … lots of what patients in other bed units miss when they are there “for storage” … I wish we could have more of this, having so much resources elsewhere too. I think it’s about resources (T)

One could think of … having a 6-8 bed unit approved for those admitted by coersion and those very psychotic, struggling hard and in need of long-lasting patient care pathways lasting two-three years. (Shifting between) being at unit and at home and gradually improving (the group confirmed) (T)

We stand there, it fills up with patients, in poor shape. And I wish so much that we had something else to offer, something else than just being here. Activity. Others get lots of resources. What we can offer to the one who is most ill, is so little. I feel it. (T)

Some talk about the long, flexible patient pathways compared to what is possible at an ordinary bed unit.

Many have been admitted several times. It is essentially different, not many units can offer that. It’s a question about resources to very large extent. A unique offer for patients admitted to the unit. An offer in another division than others (S)

#### Clinical dilemmas

Even though there are more resources, challenging situations when the employees feel highly overstretched are described. This may take place at the expence of patients or employees. The way they work comes with a cost, also for employees. Dilemmas are acknowledged; tapering off medication but also the complexity of addressing the problems that follow severe mental illness.

The person was not a danger to him-/herself or others, but very ill (Another adds:) very ill (Another adds:)… and it had something to do with resources, if everyone was without medication we would have had to have a lot more employees… to handle it… We’ve been very tired when the unit has been full and people have been very ill, wanting to die … Being a human, you are to stand in strongly, there might be some strong meetings. And people, in addition to threats and shouts, we’ve had things being thrown. We’ve had staff members being hurt, physically, acting out. Lots of sick leaves during a period of time … (talking about situation at the unit at that time:) Both needed much from the staff. (Another adds:)… and both were without medication. (Another adds:) There have been huge ethical dilemmas at least during the last admission when there was some acting out and other kind of unrest that we have had to manage (S)

For me, the strongest dilemma working there has been the open doors. We do have close monitoring, suicidal people who, as soon as you turn your back, will, and have, left and have tried to carry out attempts. Many dilemmas concerning this … (silent) …You sit (silence) sit on guard all time. Yes. (silence)… Almost following a person. And that violates what the unit is about (voluntariness) and it’s a dilemma because we do not have frames for that either… It’s some of the things that have been most exhausting … I’ve felt the anxiety of going to work, especially in evenings, weekends when we are few (few staff members) and some (patients) have close follow-up and are very suicidal ... Much responsibility in a different way (silence) (S)

Manic symptoms pose a dilemma mentioned by several.

A patient comes, tapers down, loses job, loses finances, yes, destroys finances and relations … Perhaps I would rather think that one could help, perhaps it’s not the right time to taper down. Perhaps the person needs more time. What is left after tapering off, nothing, everything is lost. Some make an exhibition of themselves. What is an exhibition of oneself for one is not necessarily the same for another. But when they afterwards say, when I get feedback on, that they are ashamed and struggle to come back for next admission, spending time and effort outside being ashamed about what happened. I feel responsibility and it affects me … I can only answer for myself, but I often wish … wish that we could do things differently (S)

A lack of adjustment and challenges with interpretation is also mentioned.

The situation might change along the way … And we do have examples where the situation has changed so much that, how to put it, we’ve not been able to adjust the treatment offered to some patients. We have examples of people being discharged from us and directly transferred to compulsory psychiatric care … (silence) (M)

Patients might feel they have to be “clever” by managing without medications, and they even feel responsible for the employees’ well-being. Employees must address the desintegration that might follow tapering down. The state of mind varies and sometimes information that seems to have been given is actually lost.

And me, personally, since I often handle medications, there are often small conversations about medication … When they tell me that they are desintegrating, losing sleep, that things happen and they lose track … It’s a medication-free unit and they feel guilty because they believe that our expectations are to taper off completely, if not, they’ll lose their place at the unit … These conversations make it clear how many times you must give information. You think you’ve informed the patient, the network. However. If the patient is participating over time, they are in different places. Information must be repeated and one must be clear. If we had frames like other units, perhaps it would not have gone so far (S)

It has recently been addressed, by a patient, who talked to all the staff … having had trouble getting back (to the unit) because of what had happened several times earlier. The person was worried fabout how we had handled it and if we (the staff) were traumatized. The person wanted to hear it in our own words, and was afraid that we felt anger toward him/her (S)

### Network

Patients’ lives are at home. The treatment must take this into consideration by including the local network, with family as an important part of this.

At the same time as providing treatment here and now, it’s a preparation for going home. It’s meant to be an improvement to bring home, develop, to work on at home. It’s this connection which is very exciting to try to make work, because it depends on the relation to the patient but also to those at home (addresses dilemma when it doesn’t work) (M)

It is perhaps also the essential part of network meetings … that everyone is allowed to say something about what is of importance for them … not only the patient … but off course the patient, too (S)

It’s the patients themselves who decide who will be part of the network in addition to the local health care therapist. Some choose the GP … some have a mental nurse in the community. Perhaps mother, father, siblings (S)

It’s important to talk about, and find out, what lies behind the anger, because it’s not the person you are really angry at. What lies beneath, unravel, to get in touch with anger can be a strength, finding the resources in every feeling. (Another adds:) Using psychoeducation, understanding what is happening both in groups, in recovery and individual conversations. And further talk about it in network if there is something that it’s important to talk more about and work on at home (S)

The participants express the possibility to be a part of a patient’s network for a long time, and also to use time to make a desicion about whether treatment at the unit is the right thing to offer or not.

One thing that differs, is regarding pathways. People are in contact with us for a long time (confirming sounds in the room). For years. We keep in touch when they are at home, more or less. I don’t think that is the case in other bed units. (Another adds:) Much of what we do are directed towards the network, they should have a therapist in their home community … and what we do are meant to be transferable to home (S)

Many find it difficult to define the specific content of the treatment, because it occurs on many levels and involves different people simultaneously.

It’s a kind of tension between what happens when the patient is admitted, what they work on intramurally, and what’s part of the individual patient’s network. That is; What is the real treatment ... at the network or at the bed unit… what we, the professionals at different levels, are involved in. And how it’s possible to be involved in these aspects at the same time. It’s a challenge to find out … A tension between being part of a network and managing a psychiatric bed unit (M)

#### Collaboration challenges

Some of the staff who cooperate with the unit give them positive feedback, but also report inconsistency regarding patient pathways. They mention, in particular, how the practice can make the therapeutic work in an acute phase difficult and how the unit’s protection of their own way of working can be challenging to handle. Some express a wish for more dialogue and an open exchange of viewpoints, not only about individual patients, but to take part in the staff’s experiences. There is an impression that the unit exists quite separated from the rest of the organized health care services, despite the focus on working with each patient’s network. However, being located inside a hospital and in dialogue (even if someone want more) with the ordinary health care services, might have positively influenced the system`s attitude (here represented by other therapists, T) towards the unit, and vice versa.

My impression is that they have been concerned about standing in it … (Elaborate on a case:) For a period the patient had to be acutely admitted … it was unclear, someone from the unit expressed ‘you are welcome back to us’. Then we experienced that they had doubts about it. It was extremely difficult to deal with, because when people are severely ill it’s essential to know whether the project will continue or not because we do not want to go in and medicate if someone has tapered down and fought for a long time. How long should we stand in it, how much should we manage and how much time should we spend: Should we support the medication free project or should we start medication again, right? It’s essential to know what, because someone must have ownership afterwards … We do not want to ruin a medication free treatment by starting medication (T)

It might be challenging when patients express distrust towards other treatment options, such as local health care services. This affects cooperation.

Some (patients) … want to talk mainly with us, because they express … the local psychiatric health service will only give me medicines. We try to talk ... So, it ends with the patients being admitted to our unit when things are difficult … Many do not feel they are met on the wish to become free of medication. (Another adds:) Some are afraid of being admitted to emergency units because some doctors are not familiar with the situation and only see the person in bad shape, of course they do become ill, thinking that something must be done … (About possibility to return) f we can handle them within our frames (open doors). Patients know this and lift themselves as much as possible to be able to come (S)

### Relations

The importance of relating to other people simply as human beings, and not only as being a patient or an employee, is a reccurrent theme. The employees’ definition of expertise in the unit is often described as more human, with a pronounced emphasis on the help seeker’s own expertise.

We are not so much experts. Of course, I have my professional background, but it’s the patients who know themselves. We must contribute to the patients getting to know themselves. (Another adds:) It’s about asking the right questions. (Another adds:) Contribute to bring things to the surface (S)

Patients of mine who have been there tell of being met in a very human way. To feel welcomed, emphasis on well-being and being an ordinary person (T)

Employees (Table 1) are well educated with clear interdisciplinary skills. Most of them have long experience from working with people with severe mental illness. Additionally, some employees have a background as peer support workers. Many of the employees express the importance of practicing being in different relationships, especially with the premise of of living a medication free life.

For people with severe mental illness it’s particularly difficult to have a space to challenge each other, talk, share experiences. We share experiences, do workout together, do things together, share new experiences. It promotes health… Being there together makes you safe in relationships, safe in…, safe common issues that allow you to go home and perhaps be safe there, with the people you have at home, at work, with the network… It’s my experience, and what I’ve heard from many people I’ve worked with, that this, relationships, can be especially difficult when you are tapering down medication. You become vulnerable, more sensitive… In my work it’s important to get into a community and make it possible to practice how to relate to others… It’s both difficult and so necessary to live a life with (psychoses) (M)

Being in a relational process involves both employees and staff and there is a neccesary dynamic between them to make it work. Some express that this is a developmental process for the unit, to let people make their own decisions. They work actively on patients’ ability to take responsibilty for their own lives.

One goal is that they should be able to ask for help but we cannot decide, really … My experience is that we have developed regarding this, in the unit. That we are able to stand in on this more than we did three years ago (M)

Co-determination is part of the empowering process.

We do get much feedback on their co-determination … Patients must be in a recovery process where our role is to support, different from what I’ve experienced before. It’s a large responsibility for the patient to be part of setting the course. For some it’s overwhelming. But when they get to know it, they say it’s very unusual. We use much time exploring the goals people have and what we must work on (S)

The process of choosing medication is described as shared decision-making in practice. One from the staff explains:

It’s the doctors who advise on, or the pace of, tapering down. I have felt, sweating, and I have been thinking ‘you dare to do that, take a look into the medical record and the medical history’. At the same time, if you are to get a chance perhaps you have to take a chance and see how it will work out. But what we have been seeing in tapering down, is that the patients themselves take more responsibility for themselves and performs in a different way, much better, when they start to taper down … instead of having something imposed on you … opposite of what they have experienced before. But of course, then they have been admitted by coercion and others have been deciding ... We also have example of patients who decide to increase (medication) (S)

However, in some cases the focus on relations is not sufficient to increase empowerment, and patients may experience disappointment, as pointed out by employees outside the unit:

They never said it, but the person thought (he/she) wasn’t the right kind of patient for them … Somehow lost the interest in handling things (him-/herself) … breaking contact with them (T)

#### Room for sharing

Many of the employees underline that there has been created a culture for sharing experiences at the unit, grounded in how they communicate in the recovery workshop.

It’s the kind of fellowship that develops because you stick together so much (M)

Being in a group where you can share, preferable in many groups, patients and staff together, patients together, set a standard, a lead that ‘here we talk’… I’ll say that it’s not only in the groups the patients talk differently together. It could have been negative if the recovery thinking had not been behind it. Somehow, we’ve … created a culture for the informal spaces too, through the recovery workshop: This is how we talk and share. A culture for the unit as a whole (M)

I think this contributes to each person’s individual process. Perhaps the person is not able to talk during lunch or in the recovery workshop, but manage to be there, listen to how other persons understand and think of what is discussed, this might be of value in the process towards accepting one’s own challenges and how to figure them out. Yes. (silence) (M)

In contrast to traditions in the hospital where it is considered important to distinguish between what one talks about with therapists and with fellow patients; sharing and listening are also welcomed in the living room at the unit. This is mostly considered positive, but may also have negative sides.

Contrary to when the structure defines where to talk and not to talk about things, we encourage to share, both in a treatment setting such as the scheduled groups, but also in the open areas, that is; in the living room. There are no rules for what to talk or not to talk about in the living room. There are conversations going on all over, really. Patients have given feedback that these conversations and exchange of experiences between them, might be as important as what the employees say (M)

A couple of times we’ve felt that it’s a little out of hand. But then it’s our job being there, to address whether it’s so wise to share so many of these thoughts in the community of the unit (S)

Many express a wanted lack of hierarchy, where sharing between patients and staff is welcomed:

We work to ensure that patients and employees participate on equal terms in a way that employees also share experiences around the topic in question. It’s like … you know your limits of what you want to share and what you are comfortable with. It feels like an arena where we try to meet on equal terms. Because it’s we, the employees, who are responsible for making sure something happens (S)

We may be a unit where employees share a lot from their lives for instance in recovery workshop … and we do it deliberately. (Another adds:) and there’s a reason for this, not to take over (breathes in) but to normalise reactions, such as grief, loss, stress, sleep problems. That it’s normal part of everyday life for everyone (S)

Working in this way can be tough for employees. The close relationship with the patients requires more from the staff, and for some this is very different from what they are used to.

I did find it difficult, in the beginning, having my meals together with patients. Being at the unit. you are together with patients all the time (mm’ing in the group). There is not much time to be alone or together with other staff. For a year’s time, I followed a patient who suffered severely, being with the person many, many times every week. Of course it is also burdensome. I think so (S)

At other units, nurses do tasks for nurses … Here it’s different and we do have (laughs) a flat structure where everyone does everything … no sharp distinction on who does what, except the senior physician … (laughs) the medical student baked buns… (laughs). The classical structure you find at other units, is dissolved … (Another adds:) It can be exhausting (S)

### Patients’ motivation

The participants underline the need to create safety without use of coercion.

We do not use coercion … we try to create safety. Making people safe rather than securing people as far as possible. I think that’s a difference. Everything we do is to be so including, open and safe that they can be there even if they become ill (M)

For me, it’s obvious, the absence (catches breath) of coercion. (The person talks about how this differs from treatment as usual). Open doors, only voluntarily admissions, even having to apply for admission. It’s about motivation … fundamentally different from what I’m used to. (Another adds:) And that there are mostly planned, and not so many acute, admissions. It’s not only when to come, but what to work on … The patients themself are to deside what to have focus on. Where am I by now and what do I need to work on … lots of content … user driven (S)

A difference here is the safe in the patient rooms that allows them to manage medications by themselves … (Another adds:) It’s not for the staff to choose (S)

The patients’ motivation for change is noted to be crucial.

Creating this culture is also affected by the fact that those who are admitted to treatment have been struggling to get there, they are strongly motivated to be exactly there. Working on their issues and working together on their recovery process. This contributes to commitment, creating a fellowship for the ones entering (silence) (M)

Those who come saying I do not want to have any contact with psychiatry. Don’t want medications, I just want to be free and then it will be fine. It doesn’t work ... You have to be willing to work on what’s difficult … There has to be motivation and you have to have some insight in that there are challenges to work on … It’s really what our treatment is about. Right. Working on what is challenging. How to understand it, see it, talk about it. Work on it (S)

## Discussion

The present study reveals that the persons working in the medication-free unit are primarily motivated by the wish to allow patients with mental illness the opportunity to live a life without medication. In attempting to capture the experienced content of “medication-free treatment”, five distinct concepts emerged, all of them interconnected by a strong focus on each patient’s unique recovery process. This is coupled with a motivation and experience of representing “something different”; a novel perspective compared to ordinary psychiatric health care.

This recovery approach leans on the individuals’ perspective on what is important, and fosters their active participation through exploration, learning and the development of new strategies. This might have had especially good conditions in Tromsø, given the hospital’s long history of employees being inspired by and engaging with psychological and social treatment approaches for psychoses. This includes systemic approaches with a strong focus on relationships and network [33–35] and the Open Dialogue approach, which advocates “less psychiatrizing forms of psychosocial support” [42], p.1)) [42–45].

While e.g. Yeisen et al. [11] express scepticism about the mandate to establish medication-free treatment, this is not found in our material. The discussion reflecting the dilemma between treatment guidelines, resources and legal framework, described by Ødegård et al. [8], is also less evident in our study. There is an overall positive attitude towards this specific treatment option, partly due the recruitment of the majority of the participants have chosen to work here and view the unit as a unique opportunity.

Our participant groups include a mix of persons with diverse professional educations, different clinical backgrounds and different relationships to the medication-free unit. This diversity sets our study apart from previous ones; while both Yeisen et al. [11] and Ødegaard et al. [8, 46] describe the participants’ attitudes where professional identity and expertise are prominently displayed when they talk about responsibility and conserns, our study highlights the patients’ expertise over the employees’.

Beyene et al. [10] noted that a holistic approach is necessary to succeed, describing this as a focus on multidisciplinary teams working with each individual patient. In our study, “holistic” is linked more to an overarching non-medical model than to the multidisciplinary approach described by Beyene et al. Haugom et al. discussed the complex collaboration between professionals and patients regarding shared decision-making [34, 35]. In our study, the emphasis on the patient’s own responsibility and autonomy in decision-making is even more pronounced.

Conceptually, defining the content and frames of the treatment is challenging. Similar to other studies on medication-free treatment [9–11], the role of the employees is both problematized and highlighted as crucial. From the informants’ narratives, it is clear that the staff seeks an alternative to “treatment as usual”, and is actively working towards this goal. Generally, the employees’ motivation for working at the unit is by many expressed through this desire to operate differently than in the more “traditional” parts of mental health care, and to find meaningfulness in their work.

The focus is on patients’ motivation and their willingness to actively engage in the recovery process, rather than merely seeking freedom from medication. This aligns with literature on recovery [16–19], and also shares similarities with the discussion raised by Haugom et al. on shared decision making [32, 33].

The relational perspective emphasized by several participants stands out as an opportunity for patients to learn and interact with others; transforming the unit into a social learning arena. This non-hierarchical approach significantly influences the working environment for employees, and employees’ sharing of their own feelings and experiences seems to be welcomed. While some find this very challenging, others view it as unproblematic. The complexity of changing professional roles and the diversity of reasons why and how this happens, have been previously described in the literature, e.g. by Nacarrow and Borthwick [47]. In light of their work, our participants seem to be developing professionalism across traditional disciplines through interdisciplinarity. However, to understand the distress some express, Fournier [48] insights are useful. She emphasizes the importance of maintaining professional boundaries and respecting their original purposes.

Medication is mentioned in the material; however, as understood from the participants’ narratives, actively tapering off medication is per se not the central aspect of thetreatment described at the unit. Attitudes towards medication more often relate to considerations around the need for psychosocial measures, focus on recovery, and general skepticism towards a “medical” or “disease-focused” model. The employees’ need to emphasize this distance is somewhat puzzling, given that Norwegian guidelines for treating psychoses strongly advocate for multidisciplinary, long-term approaches that also include psychosocial measures, and maintain an individual focus [7]. Furthermore, the unit is not a place devoid of medication, not even without psychotropics. In line with this, it appears that ‘’medication-free treatment’’ may be a proxy for prioritizing what are considered the most important treatment approaches, which does not necessarily exclude medication. This might be confusing, as mentioned also by some of the participants, and we might add, unclear; both for patients and network collaborators.

As a principle, all patients are competent to consent, and coercion is not used at the unit. This affects the selection of patients, and also relieves the staff from the ethical dilemmas and discomfort associated with coercive measures.

“Do no harm” is a well-known principle in the art of treatment, also emphasized in various guidelines ([7, 49, 50]). Several of the participants mention that they have previously been part of a system that, in their opinion, undermines people’s ability to take responsibility for themselves. There is a risk that this distancing from other part of the services might put these in a less favorable position, potentially working against the development of health services in general.

However, concerns regarding the clinical justifiability of the treatment are not frequently expressed. We anticipated such concerns, as others have expressed them [8–14]. The location of the unit within the hospital and collaboration among colleagues who know each other for a long time may have alleviated these concerns, along with the fact that medication is used when necessary. Instead, therapists not working at the unit express a desire to incorporate many of the unit’s elements elsewhere. This desire is particularly strong for the possibility of having enough time, resources to address individual needs, and a focus on psychosocial measures. Thus, a more general concern is expressed regarding resource allocation and the low prioritization of complex psychiatric patient pathways within health services, rather than the clinical justifiability of the unit. This might reflect a broader concern that the treatment approaches recognized in the medication-free unit have been removed or not implemented elsewhere, due to prioritization of shorter admissions and emergency needs.

### Strengths and limitations

The study is exploratory by nature, and we therefore decided to use an exploratory, qualitative approach. The use of group interviews addresses our assumption that group processes and interaction between the participants could bring forward valuable aspects that would increase data richness. This choice may still have introduced a risk of bias due to group-thinking, not allowing room for disagreement within the group. We were aware of this, but our evaluation was that this did not turn out to be problematic as long as divergent experiences and evaluations appeared in the groups.

We consider the overall trustworthiness of our findings to be satisfactory. The research team consisted of persons with extensive clinical backgrounds and different professional perspectives, securing credibility through checking of data collection and transcription during the process, and continuous exploratory discussions on methodology, findings, and interpretation.

The recruitment of participants was carried out in dialogue with the manager, which allows for a selection bias. However, the composition of the groups included a satisfactory variety both in professional backgrounds, age, and points of view, and during the repeated interviews, our impression was that the participants’ discussions and reflections were open and diverse. Further, we differentiated the groups (S, M and T), assuming that this might facilitate the conversation and openness. Extended triangulation was not performed, which might have reduced credibility, still the inclusion of three different groups of employees may ameliorate this risk, as may the use of two interviewers. We could also have lost some information through not including more participants not working in the unit, but we consider that the amount of data is satisfactory within the scope of the study aim.

Data dependability was secured using rigorous, detailed transcription of all interviews, and grouping of data using NVivo, which made the text easily accessible and also safeguarded the necessary distance that facilitates the analytical process. All data were accessible for the research group, and transparency was emphasized. Interpretation of the results has been discussed with a broadly composed reference group, further enhancing both credibility and confirmability of our results.

However, there is no guarantee of not falling victim to preconceptions and beliefs due to professional backgrounds and clinical experiences among the researchers. Both interviewers had primary jobs at the hospital, and many were familiar with them. This might have influenced what the participants felt free to say, and how the researchers understood what was said, due to conscious or unconscious preconceptions.

The study is based on data collected in a unique setting, and transferability is therefore somewhat limited. Still, the study brings forward general knowledge which can have transferable value also for other contexts, in particular other parts of mental health care services treating patients with severe mental illness.

## Conclusion

The findings emphasize the employees’ perception of patient autonomy as crucial. The possibility for patients to choose a life without psychotropics is strongly underscoreded, and this choice is seen as rooted more in human rights than in evidence-based studies of clinical trajectories. Surprisingly, there is less focus on tapering off medication than anticipated. The employees’ experiences reflect the therapeutic potential of treatment where focus on medication is not the dominant focus, and descriptions of psychosocial treatment elements, the recovery process, patient motivation, and non-hierarchical structures are prevalent. Enough time and resources, as well as motivational factors among employees and the network, are highlighted. In this context, the term ‘’medication-free treatment’’ may serve as a proxy for prioritizing elements other than medication. There is a perceived need to distance oneself from other part of mental health care services, accompanied by a sense of exclusivity.

Participants both within and outside the unit stress that including tailored measures in long-term treatment and focusing on recovery should not be exclusive for what is termed “medication free treatment”. As the study points out, this raises important discussions about the prioritization of health services offered in public health systems.

Overall, while the results provide valuables insights into the experiences of health professionals with medication-free treatment approaches, it should be interpreted with caution. This is an exploratory study involving a selected group of participants, where most of the participants have chosen to work in this setting and view the unit as unique, new and innovative and the remaining participants also work at the same hospital. This perspective is reflected in their attitudes and justifications. Clearly, further studies are needed to assess effects of this treatment approach.

## Supplementary Information

Supplementary Material 1.

Supplementary Material 2.

Supplementary Material 3.

## Data Availability

The dataset that supports the findings consists of in-depth qualitative interviews not publicly available for confidentiality reasons. The interview guide is available as a supplementary file.
