# Supplementary material for: Exploring the unconventional: health professionals’ experiences into medication-free treatment for patients with severe mental illness
Source: BMC Psychiatry. 2024 Nov 14;24:805. doi: 10.1186/s12888-024-06251-8 (PMC11566826; doi:10.1186/s12888-024-06251-8)
Supplement: Supplementary file 1 — Supplementary Material 1. [file 12888_2024_6251_MOESM1_ESM.pdf]

Study: Medication free treatment

(translated from Norwegian for publication. This is the edition given to group 6)

INVITATATION TO FOCUS GROUP WITH THEME OF THERAPISTS' EXPERIENCES

The study builds on interviews, self- completion forms, medical records and information from general practitioners. In addition to individual interviews with people with experiences from treatment, the study design includes focus groups with therapists.

We ask for patients' reasons for, expectations of and experiences with the treatment. And as always, we ask for questions to describe the population.

We do ask about therapists' experiences with the treatment and the follow- up by medication free treatment. It's in this context we invite you to participate.

Included groups:

Group 1,2 and 3: Staff working in the unit

Group 4: The management team in the unit

Group 5 and 6: Professionals employed other places in the hospital having experience with the unit.

Extent of time: 1,5 hour.

When: We want to conduct the interview January 2022 and will try using Whereby. The interview will be recorded (sound, not picture) and transcribed (further explained in declaration of consent).

Interviews are conducted by researcher Elisabeth Klæbo Reitan together with co-researcher Tordis Sørensen Høifødt. If you have any questions, please contact Elisabeth on phone XXXXXX.

The study has approval from Regional Committee for Medical and Health Research Ethics (REK) and from og Data Protectioan Official and funded by Northern Norway Regional Health Authority and University Hospital of North Norway. Project manager and main supervisor is Anne Høye professor at UiT The Arctic Unoversity of Norway (UiT). There are two co-supervisors Valentina Iversen, professor at Norwegian University of Science and Technology (NTNU) and Henriette Riley resaerch leader at Division of Mental Health and Substance Abuse (PHRK) and associate professor at UiT.

What to do to participate? Send research fellow a message by email elisabeth.cecilie.klaebo.reitan@unn.no or by phone XXXXXXXX as soon as possible.

Sincerely yours

Elisabeth Klæbo Reitan, research fellow Hamar, 2021
