# Supplementary material for: Exploring the unconventional: health professionals’ experiences into medication-free treatment for patients with severe mental illness
Source: BMC Psychiatry. 2024 Nov 14;24:805. doi: 10.1186/s12888-024-06251-8 (PMC11566826; doi:10.1186/s12888-024-06251-8)
Supplement: Supplementary file 2 — Supplementary Material 2. [file 12888_2024_6251_MOESM2_ESM.pdf]

Medication free treatment in Northern Norway – how is it experienced by users and practitioners?

A study of a treatment offered to patients with severe mental illness in North Norway Regional Health Authority

Translated for English for publication.

REQUEST FOR PARTICIPATION IN RESEARCH PROJECT

This is a request for you if you want to participate in a research study where we want to examine the newly established treatment offer for people with severe mental illness in The Northern Norway Regional Health Authority. We ask you because you have experiences with this treatment offer being a therapist at the unit or with patients having been in contact with the treatment unit.

The study takes place at The University Hospital of North Norway, with financial support from The North Norway Regional Health Authority.

WHAT DOES THE STUDY INVOLVE?

The study includes participation in a focus group interview with researchers. Topics are mainly your experiences in connection to this treatment offer.

POSSIBLE ADVANTAGES AND DISADVANTAGES

Possible advantages from participating in the study are that the results might lead to a better and more comprehensive knowledge of expectations to and experiences with a medication free treatment offer, which has not been established elsewhere. Results from the study may lead to the generation of new hypotheses for further research, and will be important for development of healthcare services for the group of patients you represent. You will not have any disadvantages from participating in the study, except that it may be somewhat time consuming. Times assumed is set to 1,5 hours.

VOLUNTARY PARTICIPATION AND POSSIBILITY TO WITHDRAW CONSENT

Participation in the study is voluntary. If you wish to participate, sign the declaration of consent on the final page.

Because this is a focus group interview where analyse and interpretation starts during meeting, it will be difficult to remove input after ending the session. If you do have any questions regarding the project, please contact Elisabeth Reitan.

WHAT WILL HAPPEN TO THE INFORMATION ABOUT YOU?

Information gathered about you will be used in accordance with the purpose of the study as described above. You have the right to access the information that has been gathered about you and to correct any errors.

We will record education, role, sex, age, years of experiences in general and at the unit especially. All information will be treated without name and date of birth or any directly

recognisable information. A code number links you to your data through a list of names. Only authorised personnel connected to the study will have access to this list of names and can trace the information back to you.

The project manager is responsible for day-to-day operations of the study and for ensuring that information about you is handled safely. UNN HF (the hospital) is responsible for data processing. Information about you will be anonymised or deleted no later than 2043.

It will not be possible to identify you in the results of the study when these are published.

APPROVAL

The project is approved by the Regional Committees for Medical and Health Research Ethics South-East (REK sør-øst) (2016/1708).

CONSENT FOR PARTICIPATION IN THE STUDY

Place and date   Signature participant

Participant name in capital letters

Medication free treatment in Northern Norway – how is it experienced by users and practitioners?

A study of a treatment offered to patients with severe mental illness in North Norway Regional Health Authority

My contact information is:

Name____

Date of birth____

Mobile/SMS ____

E-mail ____

Street____

Postal code/city ____

I confirm having received information about the study.

Place and date   Signature

Role in project

Kontaktinfo Elisabeth Reitan Mobil XXXXXX   elisabeth.cecilie.klaebo.reitan@unn.no
