# Supplementary material for: Exploring the unconventional: health professionals’ experiences into medication-free treatment for patients with severe mental illness
Source: BMC Psychiatry. 2024 Nov 14;24:805. doi: 10.1186/s12888-024-06251-8 (PMC11566826; doi:10.1186/s12888-024-06251-8)
Supplement: Supplementary file 3 — Supplementary Material 3. [file 12888_2024_6251_MOESM3_ESM.pdf]

Focus group interview

Introductory text: Thank you for taking part of this interview about medication free treatment and how it's experienced. Estimated time is 1,5 hours. All of you should in advance have been given information about consent and the study. (Check if everyone has signed a consent).

I (Elisabeth) will, assisted by Tordis, lead us through the questions. As mentioned in the consent and information, all of the interviews will be recorded digitally and they'll be transcribed. When information from interviews are used in analysis, they'll be deidentified so that it's not obvious that you have answered/said what you have said.

This is not part of the medication free units work, and what you say shall not have influence on your employment relationship, regardless of whether it's criticism or praise. However, since this is in a group-conversation, of course the others hear what you are saying. I hope we can make this 1,5 hour of an open conversation -as far as possible.

I will ask about four topics:

1. Background factors and working conditions (for the one interviewd)
2. Exploring questions about *the medication free treatment in Tromsø*
3. Exploring questions about *subject, ethics and possible dilemmas*
4. Exploring questions about *working with the theme medication- free-treatment*

We'll have a conversation on each topic. Since we are a small group it will work out fine.
Only for the first question, asking of who you are, we will stringently ask everyone.

BACKGROUND

Background: Name, sex, education, background (possibly professional affiliation, working experience (subject, years, time and relation to medication free unit)

Additional questions:

- Can you tell something about *why you wanted to work* at the medication free unit or *why you have had patients admitted to the unit*
- Can you say something *about everyday work at the unit* and *yours' or the employees' role at/connected to the unit?*

EXPLORING QUESTIONS - about the treatment

How does the treatment at the medication free unit differ from treatment at other units you have experience with? (Start by asking the open question before eventually asking about *what is treatment, how is it related to medication or something else.)*

How does treatment at the unit work out?

- Experiences, evaluations
- What works out well and what is difficult?

What is, in your opinion, treatment at the medication free unit?

If they don't mention the topics below, ask explicit about them;

- Patient involvement, shared decision making etc
- Collaboration on patients' treatment
- (Impact of) Time
- (Impact of) Content of the work

EXPLORING QUESTIONS – about subject, ethics and possible dilemmas

Regarding the national guidelines for treatment of psychoses and sever mental illness, we have three questions.

- How does, in your opinion, the unit for medication free treatment, relate to these guidelines?
- What is your point of view on using psychotropic medications in treatment of psychoses and bipolar disorder?
- Justifiability has been addressed and questioned in relation to medication free treatment. What do you think about justifiability?

EXPLORING QUESTIONS – about working with the theme

How has it been, and how is it by now, working with medication free treatment?
